# Causal associations between common musculoskeletal disorders and dementia: a Mendelian randomization study

**DOI:** 10.3389/fnagi.2023.1253791

**Published:** 2023-12-06

**Authors:** Jiachen Wang, Mingyi Yang, Ye Tian, Ruoyang Feng, Ke Xu, Menghao Teng, Junxiang Wang, Qi Wang, Peng Xu

**Affiliations:** ^1^Department of Joint Surgery, HongHui Hospital, Xi’an Jiaotong University, Xi’an, Shaanxi, China; ^2^Healthy Food Evaluation Research Center, West China School of Public Health and West China Fourth Hospital, Sichuan University, Chengdu, China; ^3^Department of Orthopedics, The First Affiliated Hospital of Xi’an Jiaotong University, Xi’an, Shaanxi, China; ^4^School of Health Policy and Management, Chinese Academy of Medical Sciences & Peking Union Medical College, Beijing, China

**Keywords:** musculoskeletal disorders, dementia, Mendelian randomization, genewide association studies, Alzheimer’s dementia

## Abstract

**Introduction:**

Dementia and musculoskeletal disorders (MSDs) are major public health problems. We aimed to investigate the genetic causality of common MSDs and dementia.

**Methods:**

Two-sample Mendelian randomization (MR) was used in this study. MR analysis based on gene-wide association study (GWAS) data on osteoarthritis (OA), dementia with Lewy bodies, and other MSDs and dementia types were obtained from the Genetics of Osteoarthritis consortium, IEU-open GWAS project, GWAS catalog, and FinnGen consortium. Rigorously selected single-nucleotide polymorphisms were regarded as instrumental variables for further MR analysis. Inverse-variance weighted, MR–Egger regression, weight median, simple mode, and weight mode methods were used to obtain the MR estimates. Cochran’s Q test, MR–Egger and MR-Pleiotropy Residual Sum and Outlier analysis, and the leave-one-out test were applied for sensitivity testing.

**Results:**

The inverse-variance weighted method showed that hip OA was genetically associated with a lower risk of dementia, unspecified dementia, dementia in Alzheimer’s disease, and vascular dementia. Kneehip OA was inversely associated with unspecified dementia and vascular dementia. Rheumatoid arthritis, juvenile idiopathic arthritis and seronegative rheumatoid arthritis were inversely associated with frontotemporal dementia, and rheumatoid arthritis was inversely associated with unspecified dementia. Simultaneously, ankylosing spondylitis was an independent risk factor for dementia, dementia with Lewy bodies, and dementia in Alzheimer’s disease. Sensitivity tests showed that heterogeneity and horizontal pleiotropy did not exist in these associations. The leave-one-out test showed that these associations were stable.

**Conclusion:**

We found that some MSDs were associated with the risk of dementia and provide evidence for the early detection of dementia in patients with MSDs and for the impact of inflammation on the central nervous system.

## 1 Introduction

Dementia is a syndrome that leads to a severe decline in cognitive function. As a neurodegenerative disease, it seriously affects the normal life and work of patients (Gale et al., 2018). The prevalence of dementia is 697 per 10,000 people, and the number of people with dementia doubles approximately every 5 years (Cao et al., 2020). This disease undoubtedly places a heavy burden on families and governments, with studies showing that the total cost of dementia is expected to reach US $507.49 billion by 2030 and US $1.89 trillion by 2050 (Jia et al., 2018). The most common dementia subtypes, according to clinicopathology, include Alzheimer’s dementia (ADD), vascular dementia (VaD), frontotemporal dementia (FTD), dementia with Lewy bodies (DLB), and unspecified dementia (Elahi and Miller, 2017). Although the etiologies of the various dementia types remain unclear, the risk factors for dementia mainly include advanced age, metabolic disorders, inflammation, cerebrovascular diseases, and genetic factors (Raz et al., 2016; Forloni and Balducci, 2018).

Musculoskeletal disorders (MSDs) are a serious public health problem and the most common cause of sick absence, chronic incapacity, and retirement due to ill health (Weerasekara and Hiller, 2017). An 11-year follow-up study from Norway showed that 48% of adults had at least one chronic MSDs and 20% had chronic extensive MSDs (Hagen et al., 2011). MSDs are a leading cause of pain and disability in patients, and all MSDs accounted for 6.7% of the total global disease burden in 2010 (March et al., 2014). OA was the most common MSD in 2019, reported in 527.81 million people, and the prevalence of OA is increasing annually (Hamood et al., 2021; Long et al., 2022). Similar to dementia, advanced age, genetic factors, and metabolic disorders also play an important role in MSDs, including OA, rheumatoid arthritis (RA), ankylosing spondylitis (AS), and osteoporosis (OP) (Gallo et al., 2017; Baar et al., 2018; Magnusson et al., 2023).

Both MSDs and dementia are heavy health burdens in older adults. Numerous previous studies have investigated the causal association between MSDs and dementia. According to a study by Guo et al. (2022), OA patients are at an increased risk for various cognitive impairments (Guo et al., 2022), but a retrospective cross-sectional study showed that pain interference is positively associated with dementia regardless of whether OA is present (Ikram et al., 2019). Kronzer et al. (2021) reported that the risk of ADD appears to decline over time in patients with RA (Kronzer et al., 2021). Jang et al. (2019) found that patients with AS had a higher prevalence of all-cause dementia and ADD (Jang et al., 2019). Simultaneously, some reported that sarcopenia was associated with the risk of DLB and ADD (Dost et al., 2022, 2023). However, most of these studies were retrospective, and the potential bias of residual confounding and reverse causality were unavoidable in these studies. The causality of MSDs and dementia needs to be supported by genetic evidence.

Mendelian randomization (MR) is a genetic method used to assess causal associations between an exposure or risk factor and a clinically relevant outcome (Sekula et al., 2016). MR regards a single-nucleotide polymorphism (SNP) as an instrumental variable (IV) for testing exposure because the alleles of genetic variation during meiosis are randomly assigned, and fixation of offspring genotypes occurs before exposure occurs. MR can minimize the problems of reverse causation and confounding factors (Bell et al., 2021). Because of these advantages, MR has been widely used for studying dementia and its related traits in recent years; for example, a previous MR study found that there was no genetic association between dementia and systemic lupus erythematosus and that high blood pressure was associated with a lower risk of dementia (Sproviero et al., 2021; Jin et al., 2022). In this study, we utilized publicly available gene-wide association study (GWAS) summary data for two-sample MR analysis. To our knowledge, this is the first MR study to explore the causal associations among common MSDs and various types of dementia.

## 2 Materials and methods

### 2.1 Study design

**Figure 1:**
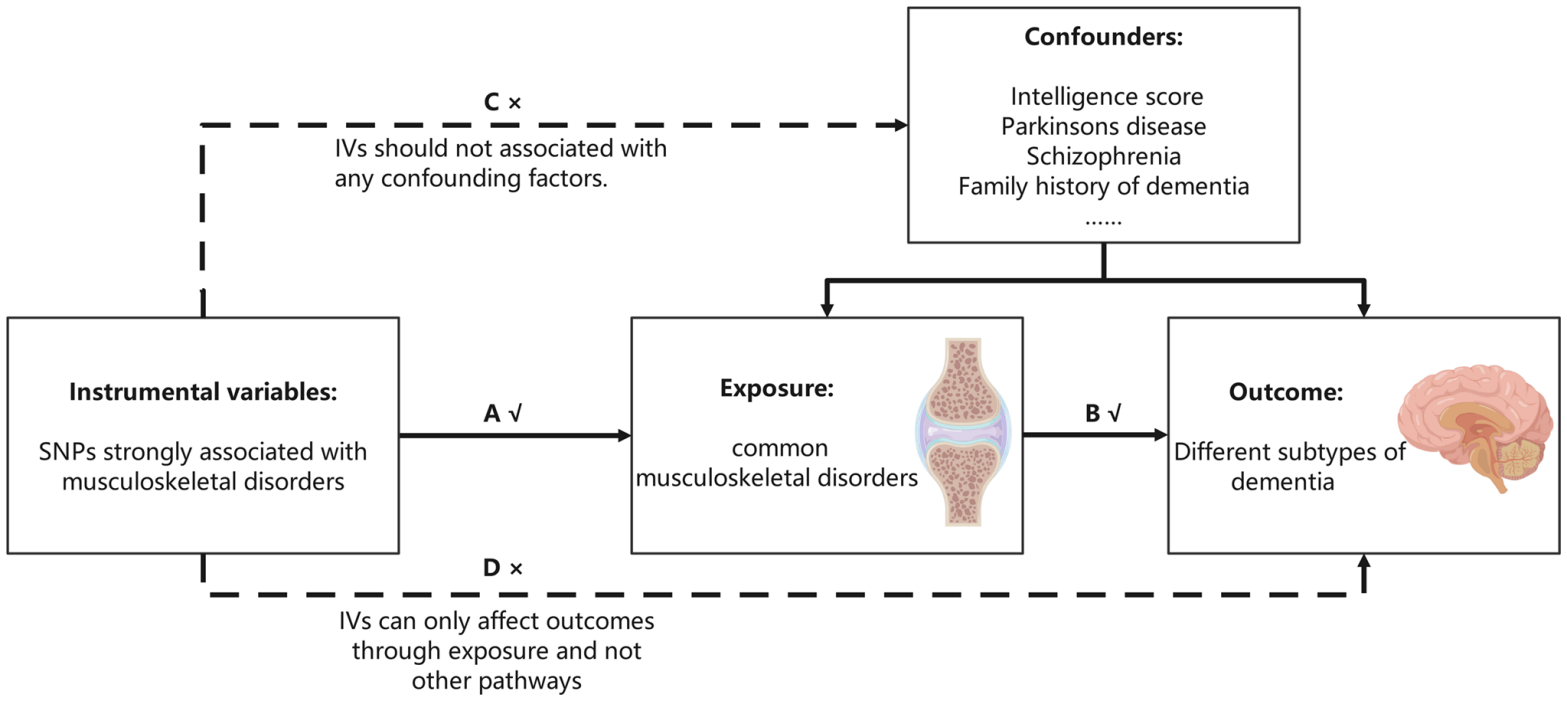
Three assumptions MR analysis must meet. The single-nucleotide polymorphisms (SNPs) strongly associated with musculoskeletal disorders were initially regarded as instrumental variables (IVs). All the IVs can only associate with dementia by musculoskeletal disorders (the solid line and ‘A’ and ‘B’ are permitted), and the selected IVs cannot associate with confounding factors or dementia directly (the dashed line and arrows ‘C’ and ‘D’ are not allowed).

We aimed to investigate the causal associations among six dementia subtypes and common MSDs. All SNPs were obtained from publicly available GWAS summary data for European populations. The MR analyses must satisfy the following assumptions: first, the screened IVs must be strongly correlated with MSDs; second, the selected IVs must not be related to confounding factors affecting the outcome; and third, IVs can only affect dementia through MSDs and not other pathways. As shown in Figure 1, the solid lines and arrows ‘A’ and ‘B’ are allowed, whereas the dashed lines and arrows ‘C’ and ‘D’ are forbidden.

R software (version 4.2.0) with the R packages “Two-sample MR” and “MR-PRESSO” was used to complete all the statistical analyses. In this study, statistical significance was set at *p* < 0.05. As this study used publicly available abstract data, no ethical approval was needed.

### 2.2 Data resource

The six dementia subgroups were regarded as outcomes in this study. The summary statistics for DLB were obtained from an independent GWAS multicenter study that included 2,591 cases and 4,027 controls of European ancestry (Chia et al., 2021). Data on dementia, ADD, VaD, FTD, and unspecified dementia were obtained from the FinnGen consortium.^1^The FinnGen project is one of the largest genetic studies aimed at improving human health by exploring genotype–phenotype correlations in the Finnish population. Our study used the 8^th^ version of the data released in December 2022, and the total sample size of this version reached 342,499. The FinnGen project relied on nationwide electronic health registers; cases were registered because they underwent major health events, including prescription drug purchases, hospitalizations, medical procedures, or deaths. The samples in FinnGen were genotyped using Illumina (San Diego, CA, United States) and Affymetrix (Thermo Fisher Scientific, Waltham, MA, USA) arrays and underwent strict quality control procedures. Sex, age, participant attributions, first 10 principal components, and genotyping batch were corrected during the analysis (Kurki et al., 2023). Information regarding the sample size and number of SNPs for the traits used in our study can be found in Supplementary Table S1.

The common MSDs were regarded as the exposure in this study. First, the summary GWAS statistic of OA was obtained from the latest published GWAS summary statistic released by the Genetics of Osteoarthritis consortium (Boer et al., 2021). We selected the clinically common ‘All OA’ (177,517 cases, 649,173 controls), ‘Kneehip OA’ (89,741 cases, 400,604 controls), ‘Knee OA’ (62,497 cases, 333,557 controls), ‘Hip OA’ (36,445 cases and 316,943 controls), and ‘Spine OA’ (28,372 cases and 305,578 controls). Second, the GWAS summary data of RA, sciatica, osteonecrosis, osteomalacia, AS, OP, fracture, and gout were obtained from the GWAS Catalog.^2^ The data on RA, AS, sciatica, osteonecrosis, osteomalacia, and OP were obtained from a generalized linear mixed model analysis of the UK Biobank (Jiang et al., 2021), which analyzed 456,348 individuals and up to 11,842,647 variants. The GWAS for fracture assessed genetic determinants of bone mineral density and fracture in 426,824 individuals with quantitative heel ultrasound (Morris et al., 2019). The GWAS for gout analyzed uric acid data from 140,000 individuals of European ancestry to obtain the genetic loci associated with gout (Köttgen et al., 2013). Third, the GWAS for osteomyelitis and main traits related to sarcopenia, including usual walking pace, physical activity, hand grip strength (left), and hand grip strength (right), was from the IEU OpenGWAS project,^3^ and age, sex, chip, and the first 10 principal component analyses were adjusted in the analysis. Finally, the GWAS summary data of muscle wasting and atrophy, seronegative rheumatoid arthritis (SNRA), seropositive rheumatoid arthritis (SPRA), meniscus derangement, lateral epicondylitis, and cervical disc disorders were obtained from the 8th version of FinnGen summary statistics. Detailed information is shown in Supplementary Table S1.

### 2.3 Instrumental variable selection

First, we selected SNPs that were strongly associated with exposure (*p* < 5 × 10^−8^, *F* > 10); and because of the limitation of the number of SNPs in physical activity, lateral epicondylitis, meniscus derangement, OP, osteomalacia, osteomyelitis, osteonecrosis, AS, spine OA and sciatica (*p* < 5 × 10^−6^) was used to screen SNPs in these traits; simultaneously, for muscle wasting and atrophy looser significant threshold (*p* < 1 × 10^−5^) was used. For the statistical power of IVs, SNPs with *F* values less than 10 were eliminated to minimize the influence of weak instrumental bias. The formula for calculating F was as follows:


$$F=R^{2}\frac{\left(N-2\right)}{\left(1-R^{2}\right)}$$

where N is the sample size and R^2^ is the genetic variation. For the calculation of genetic variation R^2^, the specific formula was as follows:


$$R^{2}=2\times EAF\times \left(1-EAF\right)\times \beta^{2}$$

where β is the allele effect value and EAF is the effect allele frequency. Simultaneously, since some row data do not have allele frequencies, the F-statistic could also be obtained using the Cragg-Donald statistic, F = β_exposure_^2^/SE_exposure_^2^ equation. Then, for the independence of SNPs, the clumping procedure with *r*^2^ < 0.001 and a window size of 10,000 kb was performed to exclude SNPs with strong linkage disequilibrium. Subsequently, PhenoscanerV2^4^ was used to remove SNPs associated with the outcomes and confounding factors. In this study, major dementia-related features and risk factors were considered confounding factors, including intelligence score, Parkinson’s disease, schizophrenia and family history of dementia (Aarsland et al., 2017; Andrews et al., 2020; Koutsouleris et al., 2022). Finally, we harmonized the exposure and outcome datasets to guarantee that the effect alleles belonged to the same alleles. SNPs that were palindromic and had intermediate allele frequencies were removed.

### 2.4 Statistical analysis

Five complementary methods, including inverse-variance weighted (IVW), MR–Egger regression, weight median, simple mode, and weight mode methods, were used to estimate the genetic causal association among 20 common MSDs and six subtypes of dementia. In this study, IVW was used as the main research method because it assumes that all IVs are valid. This method combines the Wald ratio of each IV using meta-analysis and can provide unbiased and accurate results in the absence of horizontal pleiotropy and heterogeneity (Burgess et al., 2013). The weight median method can provide relatively accurate results even when up to 50% of the selected SNPs are invalid IVs (Bowden et al., 2016). The MR–Egger method assumes that all SNPs are invalid IVs and provides estimates of causal effects. In addition, we focused on its ability to detect horizontal pleiotropy through its intercept value (Burgess and Thompson, 2017).

### 2.5 Sensitivity test

**Figure 2:**
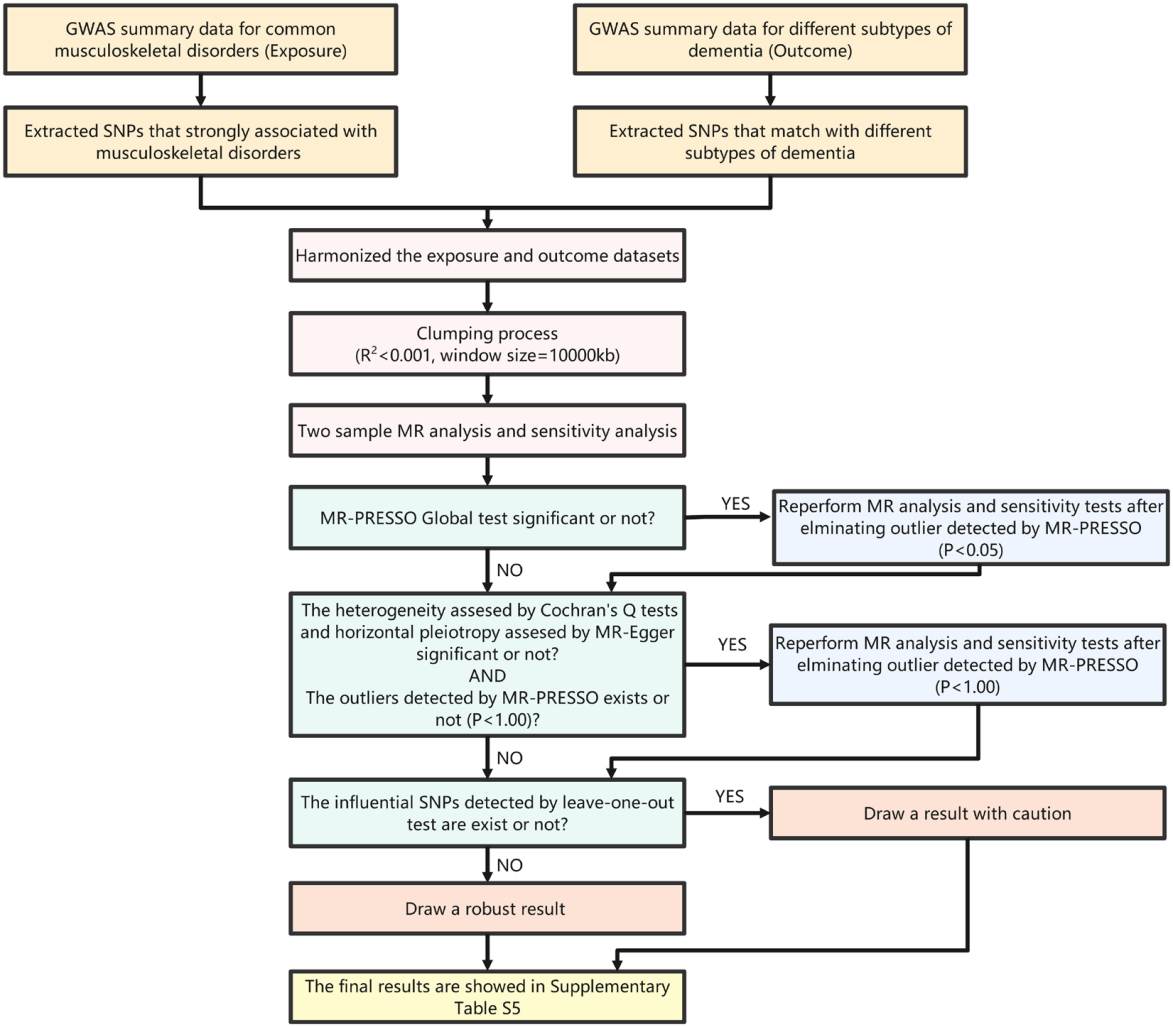
Flow chart of MR analysis.

Various methods were used to test the sensitivity of the results. First, Cochran’s Q test was performed to assess heterogeneity in the causal associations by the IVW and MR–Egger methods. Then, MR–Egger and MR-Pleiotropy Residual Sum and Outlier (MR-PRESSO) analyses were used to detect horizontal pleiotropy. Furthermore, MR-PRESSO can also detect outliers in the associations and moderate horizontal pleiotropy by outlier removal. If outliers were detected, we reperformed the MR analysis after removing the outliers. Finally, the leave-one-out test was used to test the stability of the results by removing one SNP one at a time. If an influential SNP was present, we treated the result with caution; otherwise, the result was considered robust. The process of MR analysis including the extraction of IVs and the steps of sensitivity tests is shown in Figure 2.

## 3 Results

### 3.1 Instrumental variable selection

After screening the SNPs significantly associated with MSDs and independent (r^2^ < 0.001, window size = 10,000 kb), the candidate IVs were selected. The *F* value of these candidate IVs was then calculated, and the lowest F value of these SNPs was 10.986, indicating that the influence of the weak instrumental bias on our results was low. Detailed information on the *F* values of all IVs used in this study is listed in Supplementary Table S2. Then, we eliminated the SNPs associated with confounding factors using PhenoscannerV2; the SNPs eliminated in this step are shown in Supplementary Table S3. Finally, after excluding SNPs with intermediate allele frequencies, the remaining SNPs were used for further MR analysis. The number of IVs used in the MR analysis ranged from 2 to 160 and are listed in Supplementary Table S4.

### 3.2 Causal association between osteoarthritis and dementia

**Figure 3:**
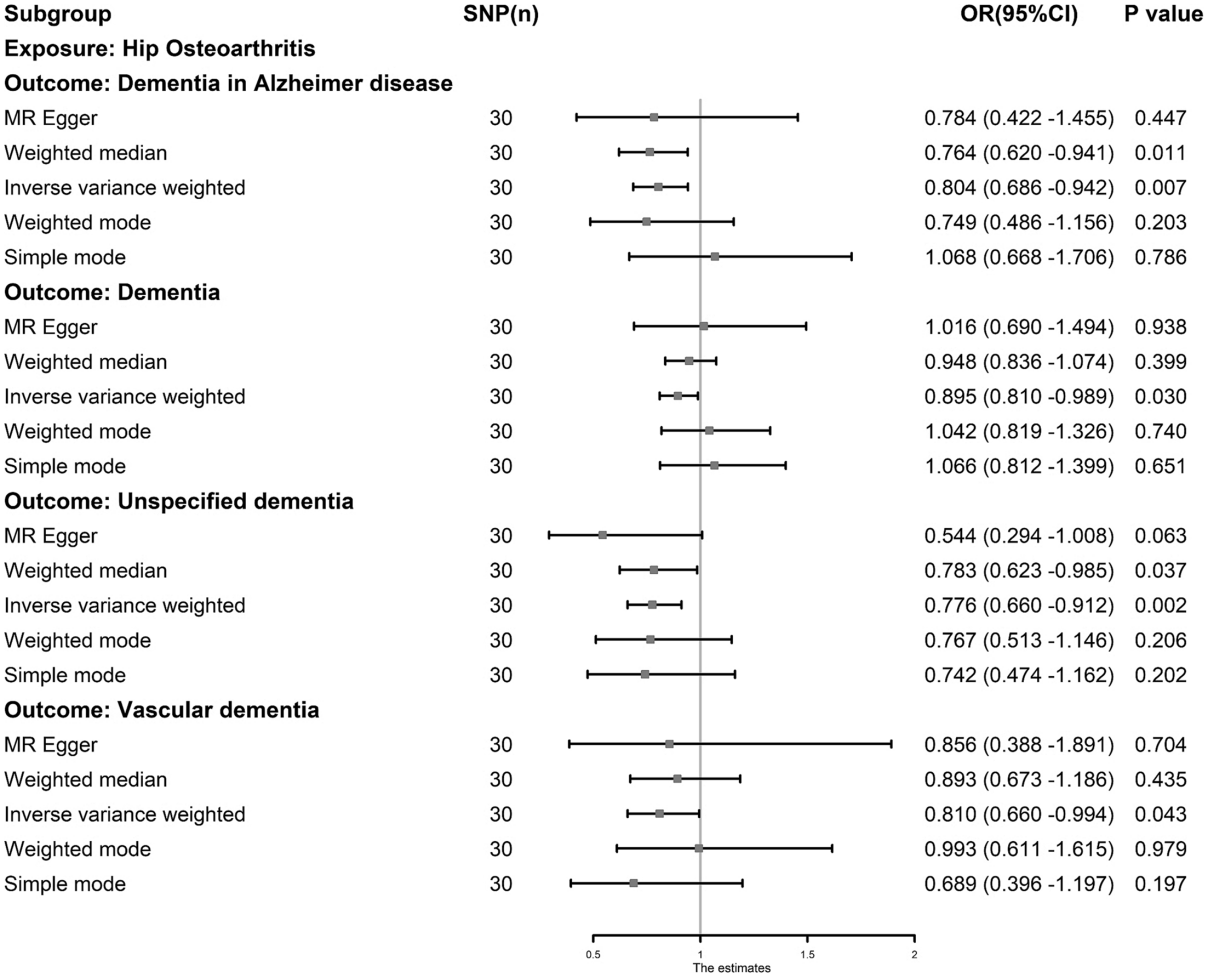
The causal association between hip osteoarthritis and dementia. Inverse-variance weighting was regarded as the major method in this study. Hip osteoarthritis was associated with a lower risk of dementia, Alzheimer’s dementia, vascular dementia and unspecified dementia.

The IVW results showed that hip OA was inversely associated with dementia (odds ratio [OR]: 0.895, 95% CI: 0.810–0.989, *p* = 0.030), unspecified dementia (OR: 0.776, 95% CI: 0.660–0.912, *p* = 0.002), ADD (OR: 0.804, 95% CI: 0.686–0.942, *p* = 0.007) and VaD (OR: 0.810, 95% CI: 0.660–0.994, *p* = 0.043) (Figure 3). Kneehip OA was inversely associated with unspecified dementia (OR: 0.754, 95% CI: 0.581–0.979, *p* = 0.034) and VaD (OR: 0.689, 95% CI: 0.509–0.934, *p* = 0.016). Simultaneously, the IVW results showed that all OA was inversely associated with dementia; however, after eliminating the outliers, all OA had a null effect on dementia (OR: 0.810, 95% CI: 0.656–1.000, *p* = 0.050). The outliers detected by MR-PRESSO are shown in Supplementary Table S5. After eliminating the outliers, sensitivity tests, including Cochran’s Q, MR–Egger, and MR-PRESSO, revealed no evidence to support the existence of heterogeneity and horizontal pleiotropy, with the exception of Knee OA and DLB (P _Egger_ = 0.019). The results of sensitivity tests are shown in Supplementary Table S6, and the results estimated by five MR methods after outlier elimination are shown in Supplementary Table S7. Funnel plots showed that the SNP distribution was symmetrical, and the leave-one-out test showed that most of the results were stable (Supplementary Figures S1, S2).

### 3.3 Causal association between ankylosing spondylitis and dementia

**Figure 4:**
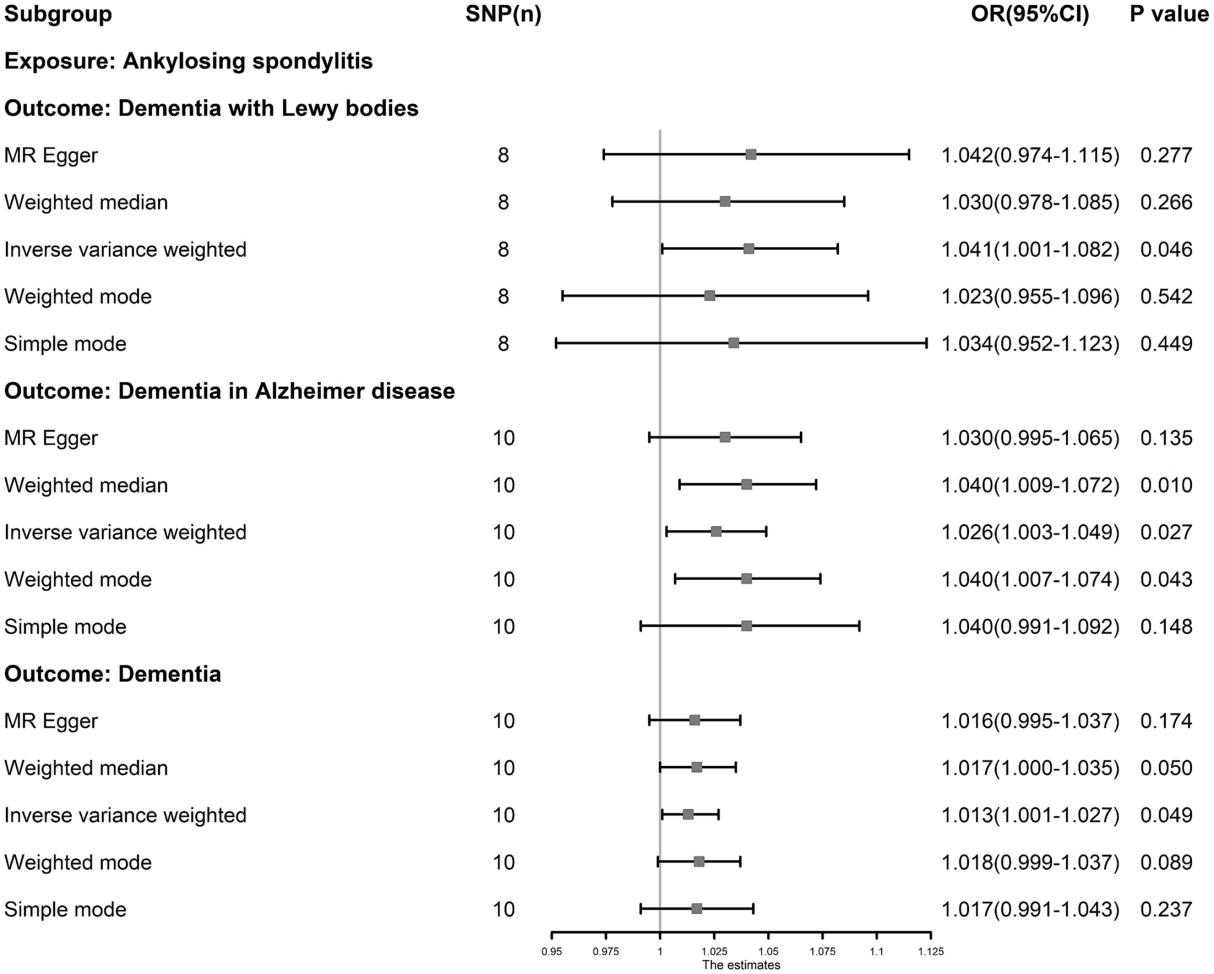
The causal association between ankylosing spondylitis and dementia. Inverse-variance weighting was regarded as the major method in this study. Ankylosing spondylitis is an independent risk factor for dementia, dementia in Alzheimer’s disease and dementia with Lewy bodies.

The IVW results showed that AS was an independent risk factor for dementia (OR: 1.013, 95% CI: 1.001–1.027, *p* = 0.049), ADD (OR: 1.026, 95% CI: 1.003–1.049, *p* = 0.027) and DLB (OR: 1.041, 95% CI: 1.001–1.082, *p* = 0.046, Figure 4). AS had a null effect on other types of dementia. MR-PRESSO analysis did not reveal any outliers in any associations. Cochran’s Q test showed no heterogeneity (*p* > 0.05), and MR-PRESSO and MR–Egger (*p* > 0.05) analyses revealed no evidence that could support horizontal pleiotropy (Supplementary Table S6). The funnel plots showed that the distortion of the SNPs was stable (Supplementary Figure S2), the leave-one-out test showed that the influential outliers exist in some associations indicating we must treat these results with caution (Supplementary Figure S1).

### 3.4 Causal association between other musculoskeletal disorders and dementia

**Figure 5:**
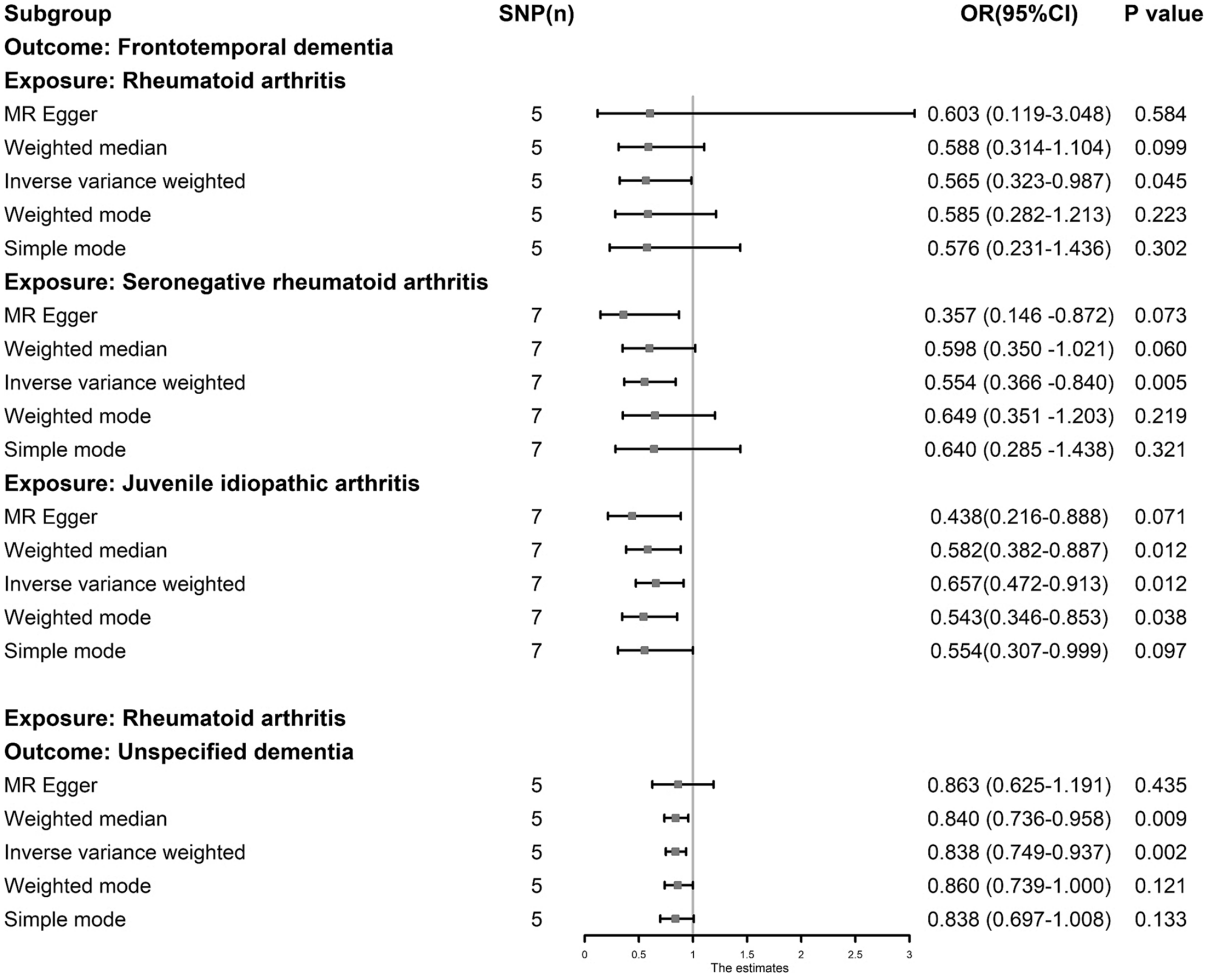
The causal association between juvenile idiopathic arthritis, rheumatoid arthritis and dementia. The results by inverse-variance weighted predict that juvenile idiopathic arthritis, rheumatoid arthritis and seronegative rheumatoid arthritis are associated with lower risk of frontotemporal dementia, and rheumatoid arthritis was inversely associated with unspecified dementia.

The IVW results showed that RA, JIA, and SNRA were inversely associated with FTD (RA, OR: 0.565, 95% CI: 0.323–0.987, *p* = 0.045; JIA, OR: 0.657, 95% CI: 0.472–0.913, *p* = 0.012; and SNRA, OR: 0.554, 95% CI: 0.366–0.840, *p* = 0.005) (Figure 5), and RA was inversely associated with unspecified dementia (OR: 0.838, 95% CI: 0.749–0.937, *p* = 0.002). The leave-one-out tests and funnel plots are shown in Supplementary Figures S1, S2. However, no causal association was observed between osteomalacia, osteomyelitis, lateral epicondylitis, osteonecrosis, sciatica, OP, lateral epicondylitis, cervical disc disorders, fracture, muscle wasting and atrophy, usual walking pace, physical activity, hand grip strength (left), hand grip strength (right) and different types of dementia (Supplementary Table S7). The outliers detected by MR-PRESSO analysis were listed in Supplementary Table S5; however, after eliminating the outliers, the original result remained stable. The results of sensitivity tests are shown in Supplementary Table S6.

## 4 Discussion

This study found broad genetic causal associations between MSDs and various types of dementia using the MR method. In our study, we found that AS was identified as an independent risk factor for dementia, DLB, and ADD. Autoimmune arthritis, including JIA, RA and SNRA, was associated with a reduced risk of FTD; furthermore, RA was inversely associated with unspecified dementia. Meanwhile, we found that OA is associated with a lower risk of different dementia subtypes. It can be concluded that some MSDs were associated with the risk factors for different subtypes of dementia.

Since the two major public health problems in middle-aged and older people are MSDs and dementia, their relationship has been widely studied. A retrospective study from South Korea showed that after adjusting for confounding factors such as sex, age, and income, patients with AS showed a higher prevalence of overall dementia and ADD than healthy controls (Jang et al., 2019). A 40-year cohort study showed that the incidence of dementia in patients with new-onset RA decreased over time (Kronzer et al., 2021). A nationwide cohort study in Taiwan showed that OA is an independent risk factor for dementia (Huang et al., 2015). There are many other studies similar to the above; however, most of these studies are based on observational evidence, which is easily affected by various confounding factors. For example, Huang et al. (2015) explained the positive causal associations between OA and dementia due to the pain and depression status of OA patients (Huang et al., 2015). Jang et al. (2019) explained the positive causal associations between AS and dementia as being caused by common risk factors such as hypertension, diabetes, and dyslipidemia (Jang et al., 2019). Furthermore, the drug treatment of MSDs also affects the causal associations between MSDs and dementia. For example, a study showed that aspirin was associated with improvements in cognitive impairment, whereas prednisone was associated with cognitive decline (Mclaurin et al., 2005). Simultaneously, a study showed that disease-modifying antirheumatic drugs (DMARDs) such as hydroxychloroquine, methotrexate and sulfasalazine have a significantly increased risk of dementia (Chou et al., 2017). To minimize the influence of the abovementioned confounding factors, this study provides new evidence for a causal association between MSDs and dementia from a genetic perspective using two-sample MR.

Our results are consistent with those of previous studies showing that AS is an independent risk factor for dementia, FTD, and ADD. AS is a chronic inflammatory rheumatic autoimmune disease. A functional magnetic resonance imaging study showed that long-term chronic inflammation caused by AS causes widespreasud changes in brain connectivity and leads to deficits in brain function (Li et al., 2017). Long-term chronic inflammation is an important feature of AS, and the activation of the NLR family pyrin domain containing 3 inflammasome (NLRP3) is an important component of the pathogenesis of AS (Kim et al., 2018). A study showed that systemic inflammation downregulates amyloid-β clearance and affects microglial morphology in an NLRP3-dependent manner, ultimately leading to cognitive impairment (Tejera et al., 2019). DLB is the second most common type of dementia after ADD and is characterized by the formation of Lewy bodies in the nerve cells of the brain, and α-synuclein is the major component of Lewy bodies (McKeith et al., 2017). Previous studies showed that the activation of the NLRP3 inflammasome is associated with the accumulation of α-synuclein aggregates, and these studies further confirmed the role of NLRP3 in amyloid β and tau driving in Alzheimer’s disease (Gordon et al., 2018; Anderson et al., 2023). Therefore, inflammation caused by AS may explain the causal relationship between AS and dementia.

In this MR analysis, no genetic association was found between RA and dementia, ADD, VAD. Previous studies on the causation between RA and dementia were complex. Some studies have shown that RA is associated with a decreased risk of dementia, whereas others have shown the opposite (Lin et al., 2018; Kronzer et al., 2021). We speculate that these contradictions may be caused by the use of DMARDs, which have been the main treatment for RA for decades. Studies have shown that all patients with RA should receive DMARD treatment as soon as possible to control symptoms and slow disease progression (Rindfleisch and Muller, 2005). A case–control study showed that patients with RA who used conventional synthetic DMARDs had a 1.63-fold higher risk of dementia than patients who did not use conventional synthetic DMARDs (Chou et al., 2017). In our study, we also found that JIA, RA, and SNRA were significantly associated with a reduced risk of FDT. FDT is a neurodegenerative disorder characterized by progressive deficits in behavior, executive function, or language that asymmetrically affect the frontotemporal lobes of the brain. Shared genetic risk factors for FDT and autoimmune diseases, including RA, have been documented (Broce et al., 2018). C9orf72 is the most common genetic background of FTD, and its causal relationship with autoimmune diseases remains uncertain. Some studies have shown that in the Finnish population, carriers of FTD C9orf72 expansion are less likely to develop autoimmune diseases than noncarriers (Katisko et al., 2018), which provides some suggestive evidence for the associations between autoimmune disease and FTD. Simultaneously, a study showed that change in the systemic inflammatory state affect the pathogenesis of FTD (Mccauley and Baloh, 2019). This MR study provides new evidence and direction for the causal associations among RA, JIA, and other autoimmune diseases and FTD through genetic methods.

This MR study also showed that OA may be related to a decreased risk of dementia. A previous study showed that OA is an independent risk factor for dementia and cognitive impairment (Guo et al., 2022), which is inconsistent with our findings. We considered several possible reasons for this difference. First, glucosamine is an important nonsurgical treatment for OA (Fransen et al., 2015), and longitudinal cohort and MR studies have shown a significant causal relationship between glucosamine and a reduced risk of dementia (Zheng et al., 2023). Meanwhile, animal experiments have shown that glucosamine can reduce inflammation in the central nervous system and protect cognitive function from damage caused by hypoxia (Lee et al., 2018). Due to the high overlap between patients with OA and glucosamine-using populations, a considerable fraction of SNPs for these two traits were concordant, which may explain the protective effect of OA on dementia observed in this study. Second, the role of inflammation in the development of dementia is complex. Although many studies suggest that inflammation has widespread neurodestructive and dementia-promoting effects (Li et al., 2017; Tejera et al., 2019), current studies have shown that microglia have both protective and destructive effects on nerves in an inflammatory environment (Enciu and Popescu, 2013). Meanwhile, apoptotic chondrocytes release RNA and activate TLR3 signaling, which is one of the important mechanisms of OA progression (Stolberg-Stolberg et al., 2022), while Bsibsi et al. (2006) showed that TLR3 is induced and activated during astrocyte inflammation, leading to the production of anti-inflammatory cytokines and ultimately mediating a comprehensive neuroprotective response (Bsibsi et al., 2006), which seems to explain the potential protective effect of OA against various subtypes of dementia. Simultaneously, our study found that there were no genetic associations between other MSDs and dementia, which is inconsistent with some previous studies. For example, a previous study reported that muscle strength was associated with the severity of dementia, and some found that sarcopenia may be more common in patients with ADD and DLB (Dost et al., 2022, 2023). We speculate that individual background factors related to sarcopenia, rather than sarcopenia itself, also contribute to the development of dementia. The studies showed that decreased muscle strength will lead to a significant increase in the incidence of mental illnesses, including anxiety and depression, and these disorders may play an indispensable role in the development of dementia (Mirza et al., 2016; Kandola et al., 2020).

To the best of our knowledge, this is the first MR study of common MSDs and dementia and has several strengths. First, the MR analysis minimized errors caused by reverse causality and confounders in the causal estimates of MSD and dementia. Second, this study used the most recent GWAS summary data, including a large sample size, and all SNPs were strictly screened to ensure the independence of the IVs and strong correlations with the exposure. Meanwhile, the lowest *F* value of the IVs was 10.986, indicating that the genetic instruments we used have strong statistical power. Finally, the use of various sensitivity tests ensured the stability of the causal estimates. Furthermore, we explained the relationship between MSDs and dementia by reviewing many previous studies. However, our study also has the following limitations. First, since all the relevant SNP data were only from studies on European populations, the results of our study should be applied to other races with caution. Second, in two-sample MR studies, sample overlap may lead to biased effect estimates, and we can only control this error by choosing other GWAS summary data. In our study, outcome data for dementia were from the FinnGen consortium, and most of the exposure data were from the Genetics of Osteoarthritis consortium and GWAS catalog. However, we were unable to find satisfactory GWAS data for SNRA, SPRA, JIA meniscus derangement, muscle wasting and atrophy, lateral epicondylitis and cervical disc disorders outside of the FinnGen consortium. We examined sample overlap of these traits with dementia, and the highest rate was 2.26% (meniscus derangement and dementia). Although this ratio is acceptable with reference to published research (Burgess et al., 2016), we still treated these results with caution. Third, we were unable to perform further subgroup analyses because of our inability to obtain detailed information and clinical stages of the participants in the GWAS. Fourth, the impact of horizontal pleiotropy on MR research is unavoidable, although we conducted a rigorous screening of the IVs used and used methods such as MR-PRESSO and MR–Egger to monitor them. Last, epigenetic problems, including DNA methylation and pathway inactivation, also affect the accuracy of MR research results to varying degrees.

## 5 Conclusion

In this study, our aim was to identify causal associations among MSDs and different types of dementia. After eliminating the outliers detected in the MR-PRESSO analysis, we found that hip OA was genetically associated with a lower risk of dementia, unspecified dementia, ADD and VaD. Kneehip OA was inversely associated with unspecified dementia and VaD; RA, JIA, and SNRA were inversely associated with FTD, and RA was inversely associated with unspecified dementia. Simultaneously, AS was an independent risk factor for dementia, ADD and DLB. This study provides new genetic evidence for the associations between MSDs and dementia and may aid in the early detection of dementia in patients with MSDs.

## Data availability statement

The original contributions presented in the study are included in the article/Supplementary material, further inquiries can be directed to the corresponding author.

## Ethics statement

Ethical approval was not required for the study involving humans in accordance with the local legislation and institutional requirements. Written informed consent to participate in this study was not required from the participants or the participants' legal guardians/next of kin in accordance with the national legislation and the institutional requirements.

## Author contributions

JiW: Conceptualization, Formal analysis, Writing – original draft, Writing – review & editing. MY: Formal analysis, Writing – review & editing. YT: Investigation, Writing – review & editing. RF: Software, Writing – review & editing. KX: Software, Writing – review & editing. MT: Investigation, Writing – review & editing. JuW: Validation, Writing – review & editing. QW: Validation, Writing – review & editing. PX: Conceptualization, Project administration, Writing – review & editing.

## Footnotes

^1^https://r8.finngen.fi/

^2^https://www.ebi.ac.uk/gwas/

^3^https://gwas.Mrcieu.ac.uk/

^4^www.phenoscanner.medschl.cam.ac.uk/
