# Corrigendum: Causal associations between common musculoskeletal disorders and dementia: a Mendelian randomization study

**DOI:** 10.3389/fnagi.2023.1362700

**Published:** 2024-01-09

**Authors:** Jiachen Wang, Mingyi Yang, Ye Tian, Ruoyang Feng, Ke Xu, Menghao Teng, Junxiang Wang, Qi Wang, Peng Xu

**Affiliations:** ^1^Department of Joint Surgery, HongHui Hospital, Xi'an Jiaotong University, Xi'an, Shaanxi, China; ^2^Healthy Food Evaluation Research Center, West China School of Public Health and West China Fourth Hospital, Sichuan University, Chengdu, China; ^3^Department of Orthopedics, The First Affiliated Hospital of Xi'an Jiaotong University, Xi'an, Shaanxi, China; ^4^School of Health Policy and Management, Chinese Academy of Medical Sciences & Peking Union Medical College, Beijing, China

**Keywords:** musculoskeletal disorders, dementia, Mendelian randomization, genewide association studies, Alzheimer's dementia

In the published article, there was an error in Figure 2 as published. The statement in Figure 2 states “GWAS summary data for six common complications of diabetes (Outcome)”. The correct statement is “GWAS summary data for different subtypes of dementia (Outcome)”. The corrected Figure 2 and its caption appear below.

**Figure 2:**
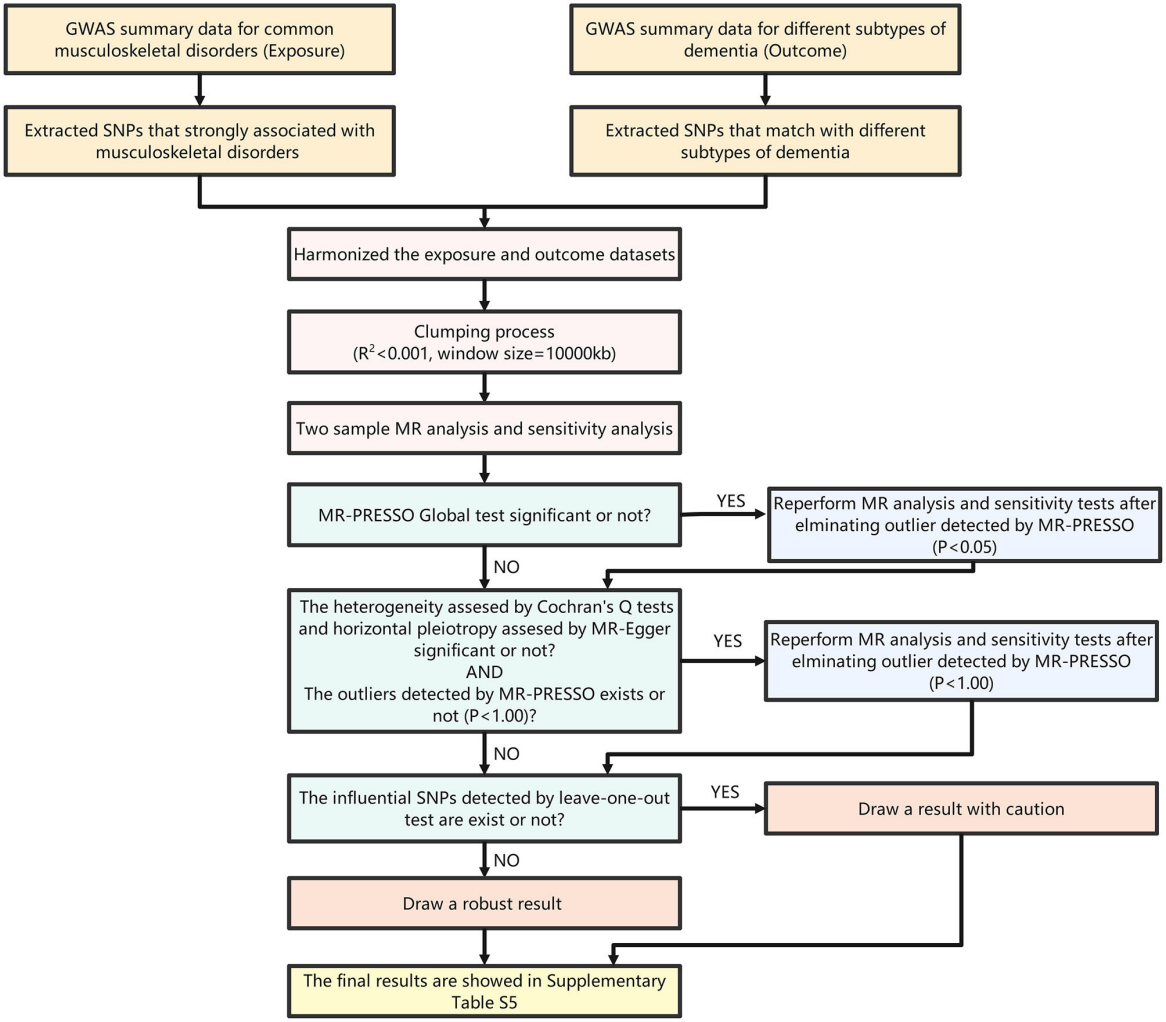
Flow chart of MR analysis.

The authors apologize for this error and state that this does not change the scientific conclusions of the article in any way. The original article has been updated.

